# Biallelic Mutations in *TMEM126B* Cause Severe Complex I Deficiency with a Variable Clinical Phenotype

**DOI:** 10.1016/j.ajhg.2016.05.021

**Published:** 2016-06-30

**Authors:** Charlotte L. Alston, Alison G. Compton, Luke E. Formosa, Valentina Strecker, Monika Oláhová, Tobias B. Haack, Joél Smet, Katrien Stouffs, Peter Diakumis, Elżbieta Ciara, David Cassiman, Nadine Romain, John W. Yarham, Langping He, Boel De Paepe, Arnaud V. Vanlander, Sara Seneca, René G. Feichtinger, Rafal Płoski, Dariusz Rokicki, Ewa Pronicka, Ronald G. Haller, Johan L.K. Van Hove, Melanie Bahlo, Johannes A. Mayr, Rudy Van Coster, Holger Prokisch, Ilka Wittig, Michael T. Ryan, David R. Thorburn, Robert W. Taylor

**Affiliations:** 1 Wellcome Trust Centre for Mitochondrial Research, Institute of Neuroscience, Newcastle University Medical School, Newcastle upon Tyne NE2 4HH, UK; 2 Murdoch Childrens Research Institute and Victorian Clinical Genetic Services, Royal Children’s Hospital, Melbourne, VIC 3052, Australia; 3 Department of Paediatrics, University of Melbourne, Melbourne, VIC 3052, Australia; 4 Department of Biochemistry and Molecular Biology, Monash Biomedicine Discovery Institute, Monash University, Clayton Campus, Melbourne, VIC 3800, Australia; 5 Functional Proteomics, SFB 815 Core Unit, Goethe-Universität, Theodor-Stern-kai 7, Haus 26, 60590 Frankfurt am Main, Germany; 6 Institute of Human Genetics, Technische Universität München, 81675 München, Germany; 7 Institute of Human Genetics, Helmholtz Zentrum München, 85764 Neuherberg, Germany; 8 Division of Pediatric Neurology and Metabolism, Department of Pediatrics, Ghent University Hospital, De Pintelaan 185, 9000 Ghent, Belgium; 9 Center for Medical Genetics, UZ Brussel, Research Group Reproduction and Genetics, Vrije Universiteit Brussel, 1090 Brussels, Belgium; 10 Population Health & Immunity Division, Walter and Eliza Hall Institute of Medical Research, Melbourne, VIC 3052, Australia; 11 Department of Medical Genetics, Children’s Memorial Health Institute, 04-730 Warsaw, Poland; 12 Metabolic Center, University Hospitals Leuven, 3000 Leuven, Belgium; 13 Neuromuscular Center, Institute for Exercise and Environmental Medicine, Texas Health Presbyterian Hospital, Dallas, TX 75231, USA; 14 Department of Pediatrics, University Hospital Salzburg, Paracelsus Medical University, 5020 Salzburg, Austria; 15 Department of Medical Genetics, Warsaw Medical University, 02-106 Warsaw, Poland; 16 Department of Pediatrics, Nutrition and Metabolic Diseases, Children’s Memorial Health Institute, 04-730 Warsaw, Poland; 17 Department of Neurology and Neurotherapeutics, University of Texas Southwestern Medical Center, Dallas, TX 75390, USA; 18 Department of Pediatrics, University of Colorado, Aurora, CO 80045, USA; 19 Department of Medical Biology, University of Melbourne, Melbourne, VIC 3052, Australia; 20 Cluster of Excellence “Macromolecular Complexes,” Goethe-Universität, 60438 Frankfurt am Main, Germany; 21 German Center for Cardiovascular Research, Partner Site RheinMain, 60590 Frankfurt, Germany

## Abstract

Complex I deficiency is the most common biochemical phenotype observed in individuals with mitochondrial disease. With 44 structural subunits and over 10 assembly factors, it is unsurprising that complex I deficiency is associated with clinical and genetic heterogeneity. Massively parallel sequencing (MPS) technologies including custom, targeted gene panels or unbiased whole-exome sequencing (WES) are hugely powerful in identifying the underlying genetic defect in a clinical diagnostic setting, yet many individuals remain without a genetic diagnosis. These individuals might harbor mutations in poorly understood or uncharacterized genes, and their diagnosis relies upon characterization of these orphan genes. Complexome profiling recently identified TMEM126B as a component of the mitochondrial complex I assembly complex alongside proteins ACAD9, ECSIT, NDUFAF1, and TIMMDC1. Here, we describe the clinical, biochemical, and molecular findings in six cases of mitochondrial disease from four unrelated families affected by biallelic (c.635G>T [p.Gly212Val] and/or c.401delA [p.Asn134Ilefs^∗^2]) *TMEM126B* variants. We provide functional evidence to support the pathogenicity of these *TMEM126B* variants, including evidence of founder effects for both variants, and establish defects within this gene as a cause of complex I deficiency in association with either pure myopathy in adulthood or, in one individual, a severe multisystem presentation (chronic renal failure and cardiomyopathy) in infancy. Functional experimentation including viral rescue and complexome profiling of subject cell lines has confirmed TMEM126B as the tenth complex I assembly factor associated with human disease and validates the importance of both genome-wide sequencing and proteomic approaches in characterizing disease-associated genes whose physiological roles have been previously undetermined.

## Main Text

Complex I deficiency is the most common biochemical phenotype observed in subjects with mitochondrial disease.^1^ It can occur as an isolated complex deficiency, where biochemical assessment of enzyme activities of other respiratory-chain components (complexes II, III, and IV) is normal, or as part of a multiple-respiratory-chain-complex deficiency with the involvement of other parts of the oxidative phosphorylation (OXPHOS) system. The latter is suggestive of a global mitochondrial defect involving, for example, mitochondrial maintenance, protein translation, or mitochondrial import. Mitochondrial complex I deficiency is phenotypically diverse, such that clinical presentations range from subacute necrotizing encephalomyelopathy (Leigh syndrome [MIM: 256000]) to pure myopathy and exercise intolerance.^1^, ^2^ In cases of isolated complex I deficiency, the genetic basis can be attributed to defects in the mitochondrial DNA (mtDNA) genes encoding seven structural subunits, in the nuclear genes encoding any of 37 other structural subunits, or in the increasing number of ancillary proteins that are responsible for faithful biogenesis and assembly of complex I. Such heterogeneity results in complicated diagnostic pipelines for clinical subjects. Massively parallel sequencing (MPS) strategies, whether in the form of whole-exome sequencing (WES)^3^ or targeted capture (e.g., Ampliseq),^4^ are proving extremely effective at establishing genetic diagnoses, particularly when mutations occur within known or candidate disease-associated genes. To date, mutations have been identified in all seven mtDNA-encoded structural subunits of complex I and 20 nuclear-encoded structural genes;^5^, ^6^, ^7^ similarly, subjects have been reported with defects in nine assembly factors.^5^ However, even after WES analysis, a significant proportion of subjects lack a genetic diagnosis—a common explanation is that their mutations affect an uncharacterized protein.^8^, ^9^ Here, we describe a cohort of six subjects who all harbor recessive mutations within the gene encoding TMEM126B, a protein recently identified as a complex I assembly factor by a proteomic study of knockdown cell lines.^10^ Complexome profiling revealed TMEM126B to be a component of the mitochondrial complex I assembly (MCIA) complex alongside proteins ACAD9, ECSIT, NDUFAF1, and TIMMDC1, thus establishing *TMEM126B* (MIM: 615533) as a candidate gene for complex I deficiency.^10^, ^11^ With access to subjects harboring putative *TMEM126B* defects, we provide functional evidence to support the pathogenicity of these *TMEM126B* variants, unequivocally establishing this gene as a cause of complex I deficiency in association with either a severe multisystem presentation in infancy or pure myopathy in later child- or adulthood. This report describes the clinical, biochemical, and molecular findings in six cases of *TMEM126B*-related mitochondrial disease and validates the importance of proteomic approaches in identifying disease-associated genes whose physiological roles have been previously undetermined.

**Figure 1:**
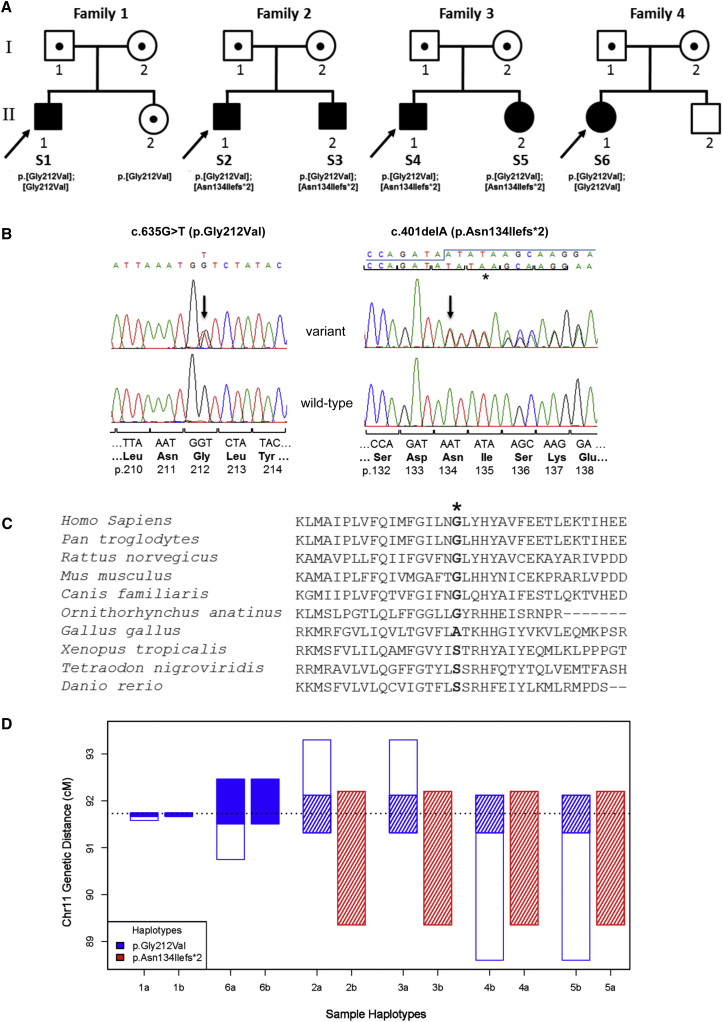
Autosomal-Recessive *TMEM126B* Variants Are Identified in Six Unrelated Subjects from Four Families Affected by an Isolated Complex I Deficiency (A) Pedigrees and genotype of affected individuals harboring *TMEM126B* variants. Subject 1 harbors a homozygous c.635G>T (p.Gly212Val) *TMEM126B* variant; his parents and unaffected sister are heterozygous carriers of this variant. Subjects 2 and 3 harbor compound-heterozygous *TMEM126B* variants—a paternal c.401delA (p.Asn134Ilefs^∗^2) variant and a maternal c.635G>T (p.Gly212Val) variant. Subjects 4 and 5 also harbor compound-heterozygous c.401delA (p.Asn134Ilefs^∗^2) and c.635G>T (p.Gly212Val) *TMEM126B* variants; carrier testing confirmed that the subjects’ mother harbors a heterozygous c.401delA (p.Asn134Ilefs^∗^2) variant, but paternal DNA was unavailable for confirmatory testing. Subject 6 harbors a homozygous c.635G>T (p.Gly212Val) *TMEM126B* variant; both her parents are carriers, and her unaffected brother does not harbor the mutation. (B) Sequencing chromatograms depict the recurrent c.635G>T (p.Gly212Val) and c.401delA (p.Asn134Ilefs^∗^2) *TMEM126B* variants, which represent the disease alleles identified in our cohort of six affected subjects. (C) Clustal Omega sequence alignment shows the evolutionary conservation of the p.Gly212 residue (marked with an asterisk). (D) Shared maternal and paternal haplotypes in the region of interest for subjects 1–6, as inferred by SHAPEIT2. Subject 1 has a ∼0.5 Mb homozygous region from 91.67 to 91.74 cM, whereas subject 6 has a ∼2 Mb homozygous region from 91.51 to 92.46 cM (blue boxes). The two Belgian sibling pairs (subjects 2 and 3 and subjects 4 and 5) share the p.Gly212Val haplotype over a ∼1.75 Mb region (91.31–92.12 cM: blue diagonal shade) and the p.Asn134Ilefs^∗^2 haplotype over a ∼4.6 Mb region (89.35–92.2 cM: red diagonal shade). Both haplotypes are shared over the 91.31–92.12 cM region. Boxed white areas represent regions shared with at least one other allele from a different family. The Polish subject 6 shares a ∼2.2 Mb (91.31–92.46 cM) p.Gly212Val haplotype region with siblings 2 and 3 and a ∼2.5 Mb (90.75–92.12 cM) region with siblings 4 and 5.

Subject 1 (family 1 subject II-1 in Figure 1A) was born to American parents without known consanguinity. He presented in childhood with pure exercise intolerance without muscle weakness. Exercise (running and swimming) caused leg fatigue, shortness of breath, and a rapid heart rate, often provoking vomiting and severe headache. Cardiology review in early adulthood showed normal electrocardiography (ECG) and echocardiography. Treadmill exercise testing caused fatigue after 2 min with a heart rate of 180 and elevated blood lactate (16 mmol/L; normal range < 2.0 mmol/L), characteristic of mitochondrial dysfunction. He had normal creatine kinase (CK) levels, and there was no pigmenturia. Physical examination remains normal at 23 years of age.

Subjects 2 and 3 (family 2 subjects II-1 and II-2, respectively, in Figure 1A) are brothers who were born to non-consanguineous parents in Belgium. They presented in their early teens with exercise-induced dyspnea (subject 2), exercise intolerance (subjects 2 and 3), and post-exertional myalgia (subjects 2 and 3). Exertion was often followed by nausea and vomiting. Now in adulthood, currently aged 40 and 37 years, respectively, subjects 2 and 3 are wheelchair bound and have significantly impaired muscle strength affecting the lower limbs, particularly hip flexion and extension. Strength in the upper limbs is normal. Forced vital capacity, cardiac ultrasound, and cognitive development are normal, and neither subject has epilepsy, neuropathy, diabetes, or hearing impairment. Subject 2 has mild visual impairment (macular and peripheral retinal pigment migration) and had mild left ventricular hypertrophy in his twenties. CK was normal, but blood lactate (2.3–3.0 mmol/L in subject 2 and 3.2–3.8 mmol/L in subject 3) and cerebrospinal fluid lactate (5.8 mmol/L in subject 3) were elevated.

Subjects 4 and 5 (family 3 subjects II-1 and II-2, respectively, in Figure 1A) are affected siblings who were born in Belgium to unrelated parents with no other children. Their father died at the age of 47 years and complained of mild exercise intolerance; their mother is alive and complains of fatigue. Subjects 4 and 5 (currently aged 33 and 30 years, respectively) presented in adolescence with fatigue, exercise intolerance, and exercise-induced nausea. No other organs are affected, although subject 5 reports gastrointestinal problems. Cardiac, ophthalmic, and nephrologic examination, intellectual capacity, and CK were normal for both subjects. Cycloergometry (for both siblings) showed very low submaximal and maximal capacity. Both subjects are able to walk but cannot ride a bike or run, and they have reported improvements following co-enzyme Q supplementation (200 mg/d).

Subject 6 (family 4 subject II-1 in Figure 1A) is female and the second child of healthy, unrelated parents living in Poland. She was born at 37 weeks of gestation with a weight of 2,150 g (third percentile [−1.88 SD]) and an Apgar score of 10. Patent ductus arteriosus and an atrial septal defect without ventricular hypertrophy were observed, and transient assisted respiration was required in the early neonatal period. At the age of 2 months, she was admitted to the hospital with very poor weight gain and vomiting, and during this period she went into cardiac arrest, attributed to gastroesophageal reflux and protracted renal failure with severe tubular acidosis (pH 7.21 [normal range = 7.35–7.43], 13.5 mmol/L NaHCO_3_ [normal range = 22.0–26.0 mmol/L], 6.0 mmol/L potassium [normal range = 3.6–5.8 mmol/L], and 124 mmol/L sodium [normal range = 136–145 mmol/L]). Progressive hypertrophic cardiomyopathy, failure to thrive, and elevated blood lactate (8.1 mmol/L) prompted suspicion of mitochondrial disease. Currently aged 6 years, she is in good general condition and has age-appropriate motor and mental development but shows chronic renal failure (stage IV) and a marked growth deficit (−5.1 SD). She requires continuous administration of erythropoietin because of anemia and is supplemented with citrate and sodium because of tubular acidosis.

Muscle and/or skin biopsy was performed for each subject, and biochemical, histochemical, and molecular investigations were undertaken (Table 1). Informed consent for diagnostic and research studies was obtained for all subjects in accordance with the Declaration of Helsinki protocols and approved by local institutional review boards.

Histochemical analysis of all subjects’ muscle biopsy revealed subsarcolemmal accumulation of mitochondria, suggestive of mitochondrial proliferation and evolving pathology of ragged-red fibers (Figure S1). Biochemical analysis of muscle respiratory-chain activities revealed a marked isolated complex I deficiency in all subjects, suggestive of a defect involving mtDNA or a nuclear-encoded protein implicit in complex I structure or assembly. The genetic basis was identified by previously described MPS strategies involving either a custom, targeted AmpliSeq panel (subjects 2 and 3) or WES (subjects 1 and 4–6) as described elsewhere.^14^, ^15^ For all cases, biallelic variants in *TMEM126B* (GenBank: NM_018480.4 and NP_060950.3) were identified—just two *TMEM126B* genotypes, either a homozygous c.635G>T (p.Gly212Val) missense variant (subjects 1 and 6) or a compound-heterozygous c.401delA (p.Asn134Ilefs^∗^2) and c.635G>T (p.Gly212Val) genotype (subjects 2–5), account for the clinical phenotype of each subject in our cohort (Table 1 and Figures 1A and 1B). Where familial samples were available from parents and unaffected siblings, these variants were found to segregate with a clinically affected status. The c.401delA (p.Asn134Ilefs^∗^2) variant is absent from dbSNP, the National Heart, Lung, and Blood Institute (NHLBI) Exome Sequencing Project Exome Variant Server (ESP6500), and the Exome Aggregation Consortium (ExAC) Browser (as of February 10, 2016). The c.635G>A (p.Gly212Val) variant is referenced in dbSNP (rs141542003) and recorded in ESP6500 (Europeans: 16/8,598 alleles [0.2%]) and the ExAC Browser (Europeans: 146/72,144 alleles [0.2%]; non-Europeans: 10/38,138 [0.02%]). No homozygous cases have been recorded (according to ExAC, ESP6500, and dbSNP data as of February 10, 2016), and subject 1 was the only individual to have rare potentially pathogenic biallelic *TMEM126B* variants in over 7,500 samples sequenced at the Institute of Human Genetics in Munich (where the c.635G>A variant was present in 15/15,134 alleles [0.1%]). Both *TMEM126B* variants have been submitted to ClinVar (see Accession Numbers).

Because the structure of TMEM126B has not been solved, in silico modeling of TMEM126B tertiary structure was performed with I-TASSER,^16^ Phyre2,^17^ and RaptorX,^18^ and although confidence was low for overall structure predictions, each tool predicted the Gly212 residue to be located within a helical domain. Glycine, the smallest amino acid and the only one without a carbon-containing side chain, is often critical within helices because it is permissive in structure and allows the helix to twist. Its substitution for a branched-chain amino acid, such as valine, is likely to affect the tertiary structure and thus compromise protein function.^19^, ^20^ This is corroborated by in silico prediction tools including SIFT,^21^ MutationTaster,^22^ and PolyPhen-2,^23^ which support a detrimental effect due to the p.Gly212Val substitution. Moderate evolutionary conservation of the Gly212 TMEM126B residue was suggested by Clustal Omega alignment of Ensembl-derived orthologs (Figure 1C).

Given that just two *TMEM126B* variants were identified in our ethnically diverse cohort of subjects (from Belgium, the United States, and Poland), we performed SNP genotyping to investigate a possible founder effect (Figure 1D and Tables S1 and S2). The most likely haplotype structure for the subjects was inferred with the SHAPEIT2 algorithm.^24^ As anticipated, there was evidence of two alleles shared by state (0.81 cM region from 91.31 to 92.12 cM) for the Belgian sibling pairs from two apparently unrelated families (subjects 2 and 3 and subjects 4 and 5). Similarly, there was a shared haplotype (1.15 cM region from 91.31 to 92.46 cM) between subjects 2 (Belgian) and 6 (Polish), and this was echoed by a 1.37 cM shared haplotype from 90.75 to 92.12 cM in an analysis involving subjects 4 (Belgian) and 6 (Polish). Together, these data support common ancestors and the c.401delA (p.Asn134Ilefs^∗^2) and c.635G>T (p.Gly212Val) variants as founder mutations. Subject 1, of European-American ancestry, was found to have a very small homozygosity-by-state (HBS) tract (0.07 cM, ∼500 kb genomic distance), but on a background suggestive of first-cousin parentage. The homozygous c.635G>T (p.Gly212Val) variant occurs within the HBS tract but is intriguingly outside the large identity-by-descent tracts shared as a result of consanguinity. This suggests that a much more distant inbreeding loop leads to this HBS tract and that the first-cousin inbreeding loop is coincidental. The sharing of haplotypes in the cohort of subjects, and that some individuals share several megabases, suggests founder events for both haplotypes; with evidence of shorter shared haplotypes, HBS, and a slightly higher frequency than that of the p.Gly212Val variant, p.Asn134Ilefs^∗^2 is likely to be the older founder event.

**Figure 2:**
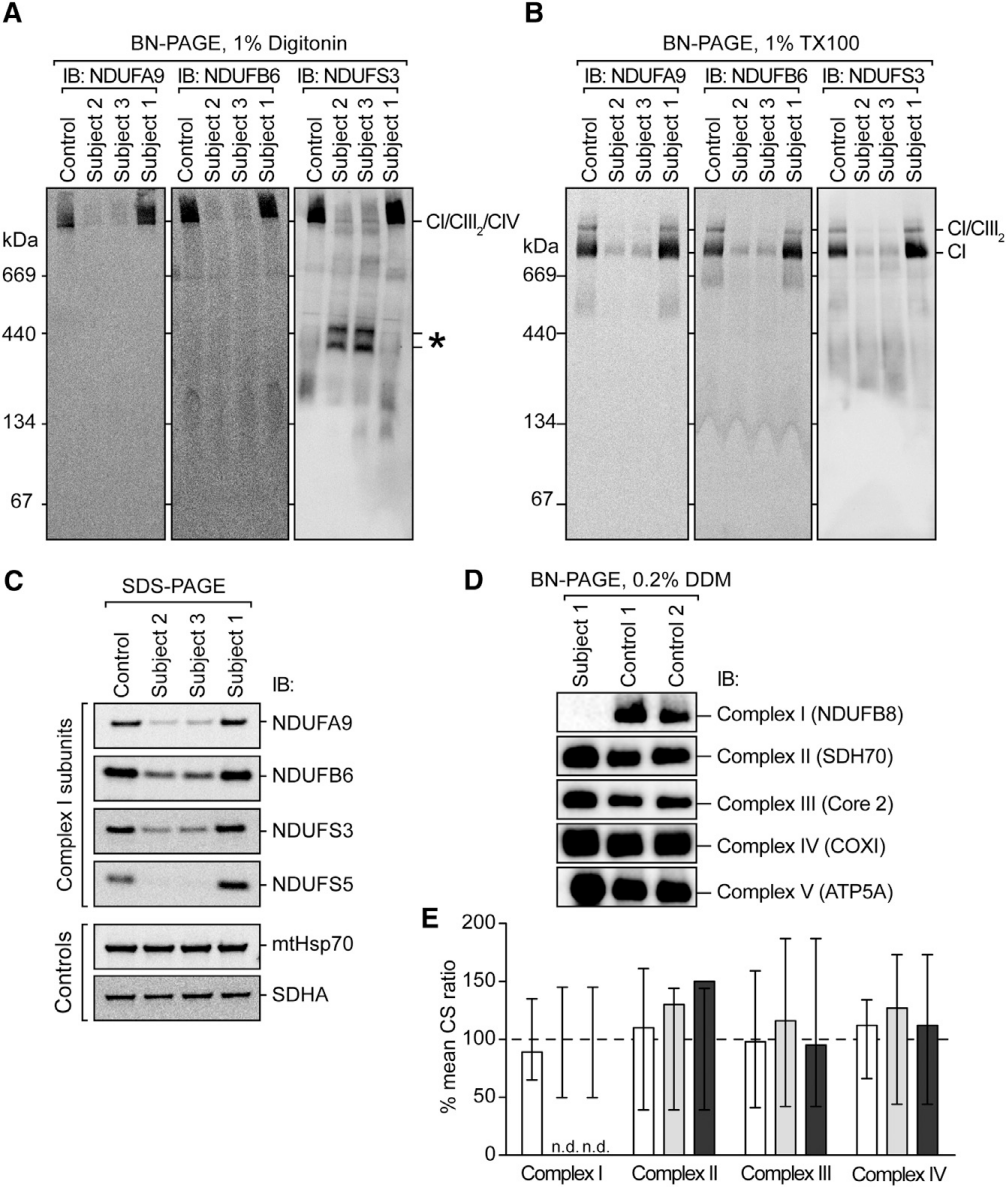
Biochemical Analysis of Subject Samples Demonstrates Tissue-Specific Complex I Deficiency (A and B) Mitochondria isolated from cultured skin fibroblasts of control subjects and subjects 1–3 were analyzed by BN-PAGE after solubilization in (A) digitonin for maintaining supercomplex interactions or (B) Triton X-100 (TX100) for isolating holo-complexes according to published methodologies.^25^ Immunoblotting was performed with antibodies as indicated. The blot probed with an antibody raised against NDUFS3 revealed the presence of additional, partially assembled complex I intermediates in the samples from subjects 2 and 3 (A, indicated by an asterisk). (C) Mitochondria were isolated as described in (A) and (B) and analyzed by SDS-PAGE. Immunoblotting was performed with antibodies against complex I subunits or control proteins as indicated. (D) Muscle samples derived from subject 1 and two control subjects were solubilized in n-dodecyl β-D-maltoside (DDM) and subjected to BN-PAGE and immunoblot analysis using antibodies directed to various OXPHOS complexes as indicated. (E) Respiratory-chain enzyme activities in fibroblast mitochondria were assayed spectrophotometrically as described^12^ and expressed as percentages of residual activity in relation to citrate synthase for subject 1 (white bars), subject 2 (light-gray bars), and subject 3 (dark-gray bars). Vertical lines represent the observed normal ranges for either 8 (subject 1) or 36 (subjects 2 and 3) normal control cell lines determined in Newcastle or Melbourne, respectively. The following abbreviation is used: ND, not detected.

Extensive functional characterization of the identified *TMEM126B* variants was undertaken in muscle and fibroblast cell lines obtained from subjects 1–3. Blue native PAGE (BN-PAGE) analysis of fibroblasts from affected subjects revealed a marked reduction of fully assembled complex I in supercomplex form (Figure 2A) or holoenzyme form (Figure 2B) in subjects 2 and 3, who harbored a truncating mutation in *trans* with a p.Gly212Val missense variant. Conversely, complex I assembly was normal in fibroblasts from subject 1, suggesting an ability to function despite the biallelic p.Gly212Val variants (Figure S1). The accumulation of subcomplexes containing NDUFS3 in subjects 2 and 3 indicates that the matrix module containing NDUFS3 is made but is unable to be added to the membrane arm. SDS-PAGE and immunoblot analysis of select complex I subunits revealed strongly reduced levels in fibroblasts from subject 2 and 3, but not subject 1 (Figure 2C). Subsequent BN-PAGE analysis of muscle from subject 1 revealed severely diminished levels of fully assembled complex I (Figure 2D).^26^ These results support a deleterious effect and recapitulate the biochemical enzyme assays in which markedly decreased complex I levels were observed in fibroblasts from compound-heterozygous subjects, whereas the fibroblasts from subject 1 retained complex I activities within the normal range (Figure 2E). Functional analysis of fibroblasts and muscle biopsy from additional individuals, notably subjects 4–6, revealed similar patterns of pathology (Supplemental Data). Two-dimensional BN-PAGE of mitochondria-enriched pellets from muscle biopsy of subjects 4 and 5 revealed a marked reduction of complex I subunits, whereas other complexes remained intact (Figure S2). Double immunofluorescence staining of fibroblasts from subjects 4 and 5 (Figure S3) or subject 6 (Figure S4) revealed decreased signal of TMEM126B directly (subjects 4 and 5) or clear evidence of reduced signal of complex I subunits in the case of subject 6 (NDUFS4 was used as a surrogate marker of complex I signal) in comparison to age-matched control subjects. Most noteworthy is the observation of a complex I biochemical defect in the cells from subject 6, who like subject 1, was homozygous for the p.Gly212Val variant yet presented much earlier in life with a more severe clinical phenotype (Table 1).

**Figure 3:**
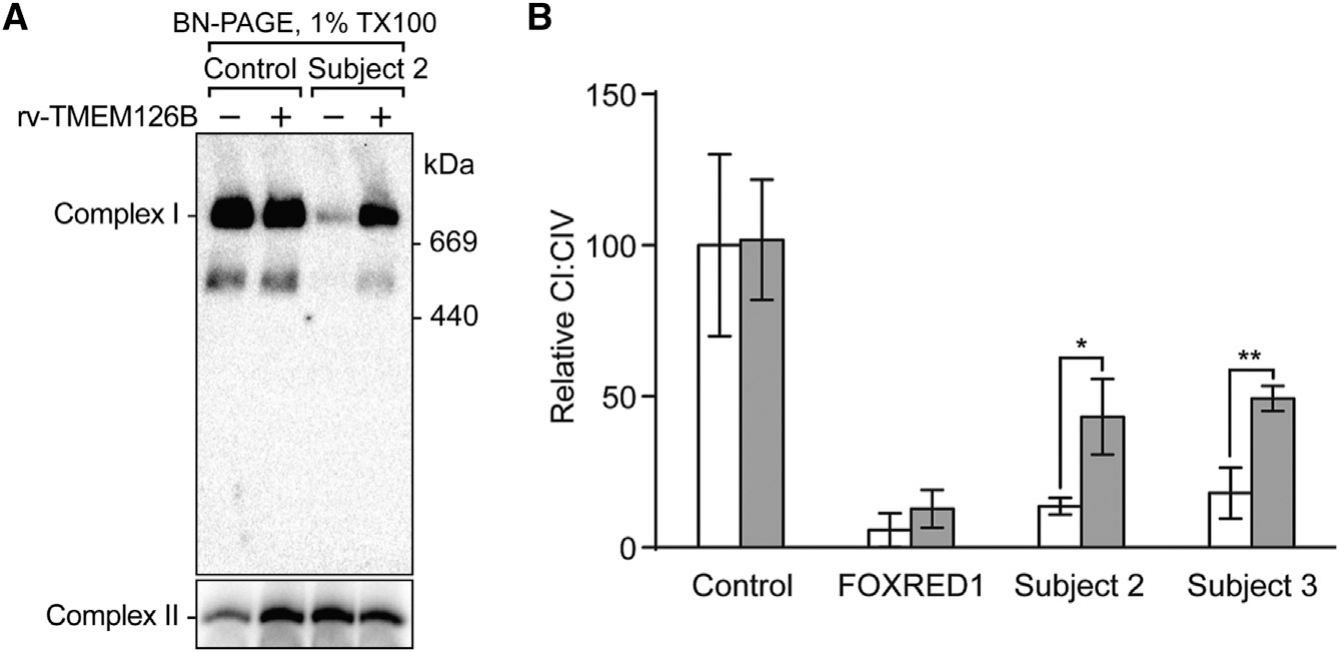
Re-expression of Wild-Type *TMEM126B* Can Lead to Increased Complex I Assembly and Activity in Subject Cells (A) Wild-type *TMEM126B* mRNA was generated by retroviral expression in control and subject 2 fibroblasts as described previously.^27^ After transduction and puromycin selection of cells, whole-cell lysates were solubilized in 1% Triton X-100 and analyzed by BN-PAGE and immunoblotting using antibodies against NDUFA9 (complex I) and SDHA (complex II) as a loading control. (B) Wild-type *TMEM126B* mRNA was generated by lentiviral expression, and activities of complexes I and IV were assessed by enzyme dipstick analyses as described previously.^4^ Barplots show complex I (CI) activity, normalized by complex IV (CIV) activity in control and subject fibroblasts,^28^ after transduction with (gray bars) and without (white bars) wild-type *TMEM126B* mRNA. Data shown are means of three independent transfections ± SEM. ^∗^p < 0.05, ^∗∗^p < 0.01.

To provide further evidence that *TMEM126B* mutations are causative, we performed cellular rescue with *TMEM126B* variant 1 (GenBank: NM_018480.4) essentially as described previously^4^ (Figure 3). Retroviral-mediated expression of *TMEM126B* in subject 2 fibroblasts largely restored the levels of assembled complex I (Figure 3A). In addition, after lentiviral-mediated expression of *TMEM126B*, enzyme activities were significantly increased in fibroblasts re-expressing *TMEM126B* from subjects 2 and 3, whereas fibroblasts from a healthy control or subject with recessively inherited, pathogenic *FOXRED1* variants (described previously^28^) showed no increased activity (Figure 3B).

**Figure 4:**
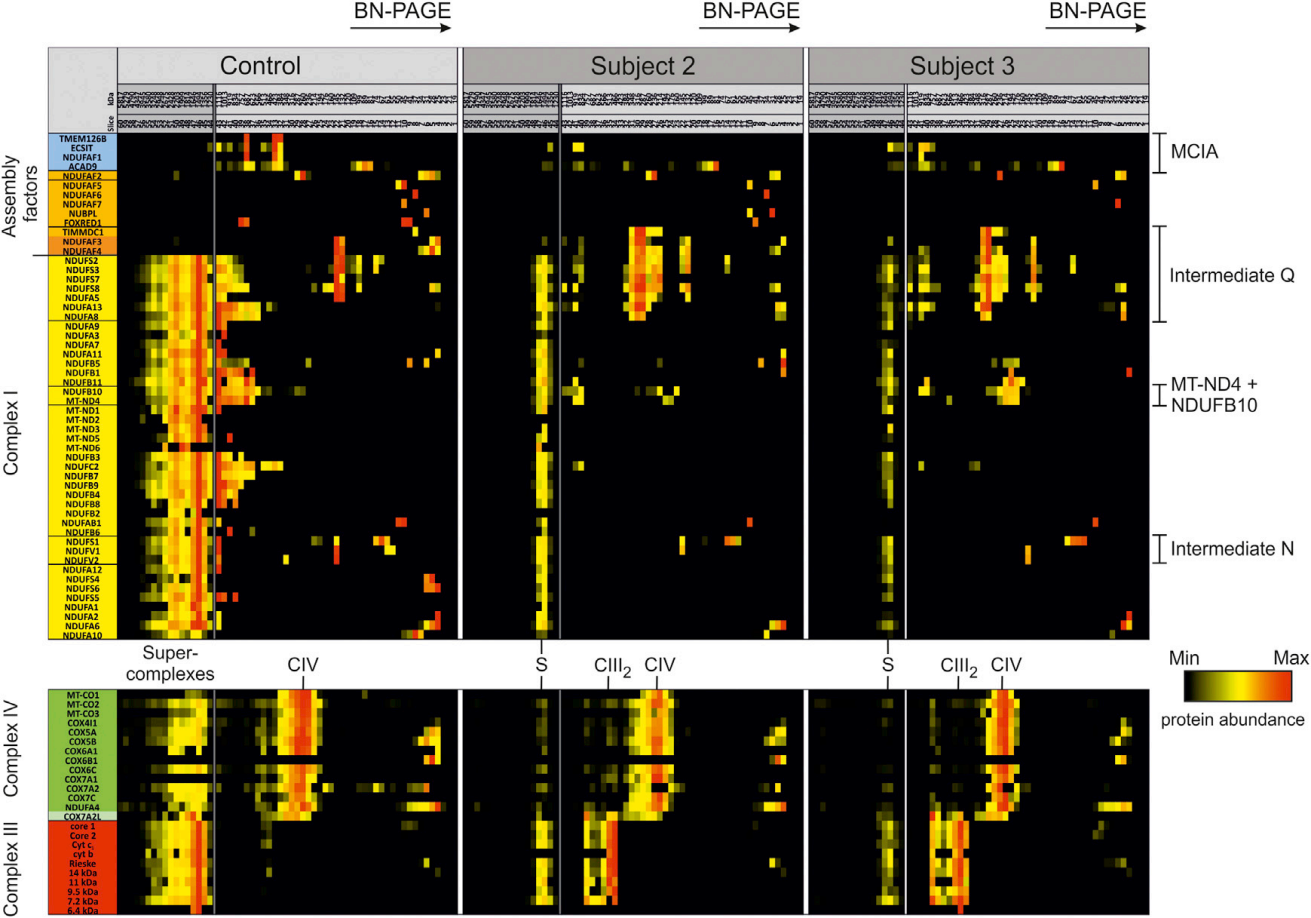
Complexome Profiling of Fibroblasts from Subjects 2 and 3 Identifies Stalled Complex I Assembly Intermediates Prior to mitochondrial isolation, skin fibroblasts were cultured for 48 hr in medium supplemented with galactose as a carbon source. Mitochondrial protein complexes were solubilized with digitonin and separated by BN-PAGE.^30^ Native gels were fixed and stained with Coomassie and cut into 60 equal fractions; proteins were digested with trypsin and analyzed by quantitative mass spectrometry. For direct comparison of protein-abundance profiles in control and affected subjects, intensity-based absolute quantification values^31^ calculated by MaxQuant proteomics software^32^ were normalized to the maximum over datasets (left part of each sample). Less abundant complex I assembly intermediates were normalized to the maximum within the mass region below 1,200 kDa (right part of each sample) for enabling better visualization within a heatmap. The native masses of gel slices were calibrated by exponential regression using positions of the human OXPHOS complex in the gel.^33^ The left lane indicates assembly factors (orange), MCIA components (blue), and structural subunits of complex I (yellow), complex III (red), and complex IV (green). Abbreviations are as follows: MCIA, mitochondrial complex I assembly complex; CIII_2_, complex III dimer; CIV, complex IV; and S, supercomplex containing complex I, a dimer of complex III, and one to four copies of complex IV.

TMEM126B was identified as a component of the MCIA complex, which also comprises the previously characterized assembly factors ACAD9, ECSIT, and NDUFAF1.^10^, ^29^ To gain deeper insight into the molecular consequences of the subjects’ *TMEM126B* variants, we analyzed complex I assembly by complexome profiling. As visualized in a heatmap (Figure 4), profiles of protein abundance confirmed a severe complex I assembly defect in fibroblasts from subjects 2 and 3 and a concomitant increase in the amount of free complex III. Prominent accumulation of a stalled assembly intermediate containing subunits of the Q module and assembly factors NDUFAF3, NDUFAF4, and TIMMDC1 was observed, reflecting the 315 kDa subcomplex reported by *TMEM126B* siRNA experiments in 143B cells.^29^ Consistent with TMEM126B-knockdown cells,^10^ subject mitochondria formed a ∼200 kDa subcomplex containing ND4 and NDUFB10, indicating that parts of the membrane arm can be assembled without a complete MCIA complex. Recent work on TIMMDC1-knockdown cell lines has suggested that the membrane protein TIMMDC1 connects the pre-assembled membrane subcomplex via interaction with MCIA components.^11^, ^29^ In subject mitochondria, only low levels of the membrane subcomplex were found in the large assembly intermediate of 830–1,000 kDa, reflecting an inefficient connection of the Q module and membrane modules. In contrast to TMEM126B-knockdown cells,^10^ the subject cell lines showed markedly decreased levels of ECSIT, ACAD9, and NDUFAF1, the remaining MCIA components, together with complex I subunits in a mass region between ∼800 and 950 kDa. The last part of the assembly sequence uses assembly factor NDUFAF2 and the preassembled NADH dehydrogenase module (N module) to complete complex I.^34^, ^35^, ^36^ Fibroblasts from both affected subjects showed low amounts of preformed N module intermediates, indicating that assembly of the membrane part might control N module assembly such that it does not accumulate in the affected cells. NDUFAF2 was not detected in a complex with N module subunits but shifted from ∼260 kDa in control cells to ∼230 kDa in affected cells. It is known that once complex I assembly is completed, all assembly factors dissociate and leave a fully assembled complex I.^37^ In cells from subjects 2 and 3, we identified minor amounts of ECSIT and ACAD9 still bound in the native mass region corresponding to supercomplex S1, containing complexes III and IV. It remains to be further determined whether these large assembly intermediates are already part of a supercomplex or co-migrate in this high-molecular-mass region. Another assembly factor, FOXRED1, recently identified to exhibit a function in late-stage assembly,^25^ was identified as co-migrating with the large assembly intermediate of ∼700–800 kDa in control fibroblasts. In subjects 2 and 3, FOXRED1 could be detected only at the electrophoretic front and not in a complex with complex I subunits, suggesting that the MCIA complex is a prerequisite for binding and function in the large intermediate.

Tissue specificity is a common phenomenon in OXPHOS disorders,^38^ but this feature is particularly striking with the *TMEM126B* mutations we describe here. Five of the six subjects have relatively mild symptoms, confined mostly to myopathy, and all have normal cognitive development despite having a severe complex I defect in muscle and the fact that TMEM126B appears to be a ubiquitous complex I assembly factor. All tissues studied from subjects with *TMEM126B* mutations showed some residual complex I assembly, and the threshold level of complex I activity required by any tissue most likely depends on factors such as variation in the amounts of subunits and assembly factors, plus variation in protein turnover rates and basal and peak energetic demands. Inter-individual differences were also apparent—all subjects except subject 1 had a marked complex I defect in skin fibroblasts. We note that subject 6’s congenital heart defects, low birth weight, and episode of acute dehydration most likely contributed to the severity of her symptoms. However, subjects 1 and 6 are both homozygous for the p.Gly212Val variant, but fibroblast complex I activity was normal in subject 1 and deficient in subject 6. Hence, the variation in outcomes also most likely relates to a combination of the severity of different mutations and as yet unknown genetic modifiers affecting the biochemical and clinical phenotypes. It is also possible that the alternative TMEM126B isoforms arising through alternative splicing of the *TMEM126B* mRNA transcripts might affect the clinical phenotype. The possibility of pathogenic variants within the *TMEM126B* paralog, *TMEM126A*, was excluded in subject 6 by analysis of the WES dataset; moreover, optic atrophy is a discriminatory feature in cases of *TMEM126A* pathology, and this individual has normal visual acuity.

Characterization of TMEM126B after proteomic screening and subsequent application of diagnostic MPS strategies has resulted in the diagnosis of six subjects from four families affected by *TMEM126B*-related mitochondrial disease. Our subjects suggest that a late-onset myopathic phenotype is the predominant clinical phenotype associated with *TMEM126B* defects. Functional experimentation including lentiviral rescue of subject fibroblasts establishes *TMEM126B* as the tenth complex I assembly factor associated with human disease, and this gene should be considered in the molecular genetic workup of subjects with biochemical evidence of an isolated complex I deficiency, particularly in European populations.

## Figures and Tables

**Table 1:** Biochemical and Clinical Findings in Individuals with *TMEM126B* Variants

**Subject Details**		***TMEM126B* Variants**	**OXPHOS Activities in Skeletal Muscle**				**Clinical Features**		
**ID**	**Sex**	**cDNA (GenBank:****NM_018480.4****), Protein (GenBank:****NP_060950.3****)**	**RCC**	**Mean Enzyme Activity**	**Absolute Values**	**Control Mean (Reference Range)**	**Age at Onset**	**Clinical Course**	**Other Clinical Features and Relevant Family History**
Subject 1^a^	male	c.[635G>T];[635G>T], p.[Gly212Val];[Gly212Val]	I	36% (↓)^b^	1.8	5.0 ± 0.8 (n = 28)	8 years	alive at 21 years	exercise intolerance, unable to perform sustained aerobic exercise, normal strength, normal ECG and echocardiography, normal resting lactate, normal CK
			II	210% (↑↑)	4.2	2.0 ± 0.6 (n = 44)			
			III	219% (↑↑)	23.6	10.8 ± 2.3 (n = 29)			
			IV	218% (↑↑)	8.5	3.9 ± 1.5 (n = 44)			
			CS	196% (↑↑)	24.1	12.3 ± 2.7 (n = 44)			
Subject 2^c^	male	c.[401delA];[635G>T], p.[Asn134Ilefs^∗^2];[Gly212Val]	I	48% (↓)^b^	14	29 ± 13 (n = 30)	12 years	alive at 39 years, wheelchair bound	exercise intolerance, muscle weakness in lower limbs and pelvis, normal echocardiography, mild basal increases of lactate, normal CK, normal intelligence, retinitis pigmentosa
			II	138%	47	34 ± 14 (n = 30)			
			III	ND	ND	96 ± 31 (n = 30)			
			IV	82%	137	167 ± 58 (n = 30)			
			CS	237% (↑↑)	412	174 ± 70 (n = 30)			
Subject 3^c^	male	c.[401delA];[635G>T], p.[Asn134Ilefs^∗^2];[Gly212Val]	I	14% (↓↓)^b^	4	29 ± 13 (n = 30)	10 years	alive at 36 years, wheelchair bound	clinically affected sibling of subject 2, exercise intolerance, muscle weakness in lower limbs and pelvis, normal echocardiography, mild basal increases in lactate, normal CK, normal intelligence, no retinitis pigmentosa
			II	179% (↑)	61	34 ± 14 (n = 30)			
			III	ND	ND	96 ± 31 (n = 30)			
			IV	103%	172	167 ± 58 (n = 30)			
			CS	281% (↑↑)	489	174 ± 70 (n = 30)			
Subject 4^a^	male	c.[401delA];[635G>T], p.[Asn134Ilefs^∗^2];[Gly212Val]	I	10% (↓↓)^b^	3	29 ± 13 (n = 30)	8 years	alive at 32 years	exercise intolerance and fatigue
			II	253% (↑↑)	86	34 ± 14 (n = 30)			
			III	172% (↑)	165	96 ± 31 (n = 30)			
			IV	126%	210	167 ± 58 (n = 30)			
			CS	201% (↑↑)	350	174 ± 70 (n = 30)			
Subject 5^a^	female	c.[401delA];[635G>T], p.[Asn134Ilefs^∗^2];[Gly212Val]	I	10% (↓↓)^b^	3	29 ± 13 (n = 30)	15 years	alive at 29 years	clinically affected sibling of subject 4, exercise intolerance and fatigue
			II	288% (↑↑)	98	34 ± 14 (n = 30)			
			III	129%	124	96 ± 31 (n = 30)			
			IV	238% (↑↑)	398	167 ± 58 (n = 30)			
			CS	259% (↑↑)	451	174 ± 70 (n = 30)			
Subject 6^a^	female	c.[635G>T];[635G>T], p.[ Gly212Val];[Gly212Val]	I	17% (↓↓)^b^	3	17 ± 8 (n = 15)	2 months	alive at 5.5 years	multiorgan involvement manifesting in infancy (respiratory failure, cardiomyopathy, and renal acidosis), severe growth failure, chronic renal insufficiency, elevated serum lactate
			II	135%	13	10 ± 3 (n = 15)			
			III	64%	58	90 ± 52 (n = 15)			
			IV	82%	10	12 ± 9 (n = 15)			
			CS	228% (↑↑)	458	200.9 ± 48.5 (n = 15)			

For subject 1, respiratory-chain enzyme activities are expressed as U/min/g wet weight.^12^ For subjects 2–6, enzyme activities are expressed as nanomoles of substrate/min/mg protein.^13^ The following abbreviations are used: ↓, decreased; ↓↓, markedly decreased; ↑, increased; ↑↑, markedly increased; ECG, electrocardiography; and ND, not determined.

a Investigated by WES.

b Below the normal range.

c Investigated by targeted gene analysis (AmpliSeq capture or carrier testing).

## Accession Numbers

The accession numbers for variants c.401delA and c.635G>T are ClinVar: SCV000280578 and SCV000280579, respectively.

## Web Resources

Clustal Omega, http://www.ebi.ac.uk/Tools/msa/clustalo/
dbSNP, http://www.ncbi.nlm.nih.gov/SNP/
Ensembl, http://www.ensembl.org/
ExAC Browser, http://exac.broadinstitute.org
I-TASSER, http://www.zhanglab.ccmb.med.umich.edu/I-TASSER
NHLBI Exome Sequencing Project (ESP) Exome Variant Server, http://evs.gs.washington.edu/EVS/
OMIM, http://www.omim.org
Phyre2, http://www.sbg.bio.ic.ac.uk/phyre2/
PolyPhen-2, http://genetics.bwh.harvard.edu/pph2/
RaptorX, http://raptorx.uchicago.edu
RefSeq, http://www.ncbi.nlm.nih.gov/refseq/
SHAPEIT2, https://mathgen.stats.ox.ac.uk/genetics_software/shapeit/shapeit.html
SIFT, http://sift.jcvi.org/

## Supplemental Data

Document S1. Figures S1–S4

Table S1. Phased Haplotypes Haplotypes for subjects 1–6 were inferred by the SHAPEIT2 algorithm. Shared regions are colored in the same shade, where blue represents the haplotype carrying the p.Gly212Val variant, and red is the haplotype carrying the p.Asn134Ilefs^∗^2 variant.

Table S2. Shared Haplotype Coordinates Haplotype sizes and coordinates for subjects 1–6 were inferred by the SHAPEIT2 algorithm, where Iab_eq_Jab represents both alleles shared across individuals I and J, Ib_eq_Jb is haplotype b shared across individuals I and J, etc.

Document S2. Article plus Supplemental Data
