# Supplementary material for: Biallelic Mutations in TMEM126B Cause Severe Complex I Deficiency with a Variable Clinical Phenotype
Source: Am J Hum Genet. 2016 Jun 30;99(1):217–27. doi: 10.1016/j.ajhg.2016.05.021 (PMC5005451; doi:10.1016/j.ajhg.2016.05.021)
Supplement: Document S1. Figures S1–S4 [file mmc1.pdf]

The American Journal of Human Genetics, Volume *99*

Supplemental Data

Biallelic Mutations in *TMEM126B* Cause Severe Complex I Deficiency with a Variable Clinical Phenotype

Charlotte L. Alston, Alison G. Compton, Luke E. Formosa, Valentina Strecker, Monika Oláhová, Tobias B. Haack, Joél Smet, Katrien Stouffs, Peter Diakumis, Elżbieta Ciara, David Cassiman, Nadine Romain, John W. Yarham, Langping He, Boel De Paepe, Arnaud V. Vanlander, Sara Seneca, René G. Feichtinger, Rafal Płoski, Dariusz Rokicki, Ewa Pronicka, Ronald G. Haller, Johan L.K. Van Hove, Melanie Bahlo, Johannes A. Mayr, Rudy Van Coster, Holger Prokisch, Ilka Wittig, Michael T. Ryan, David R. Thorburn, and Robert W. Taylor

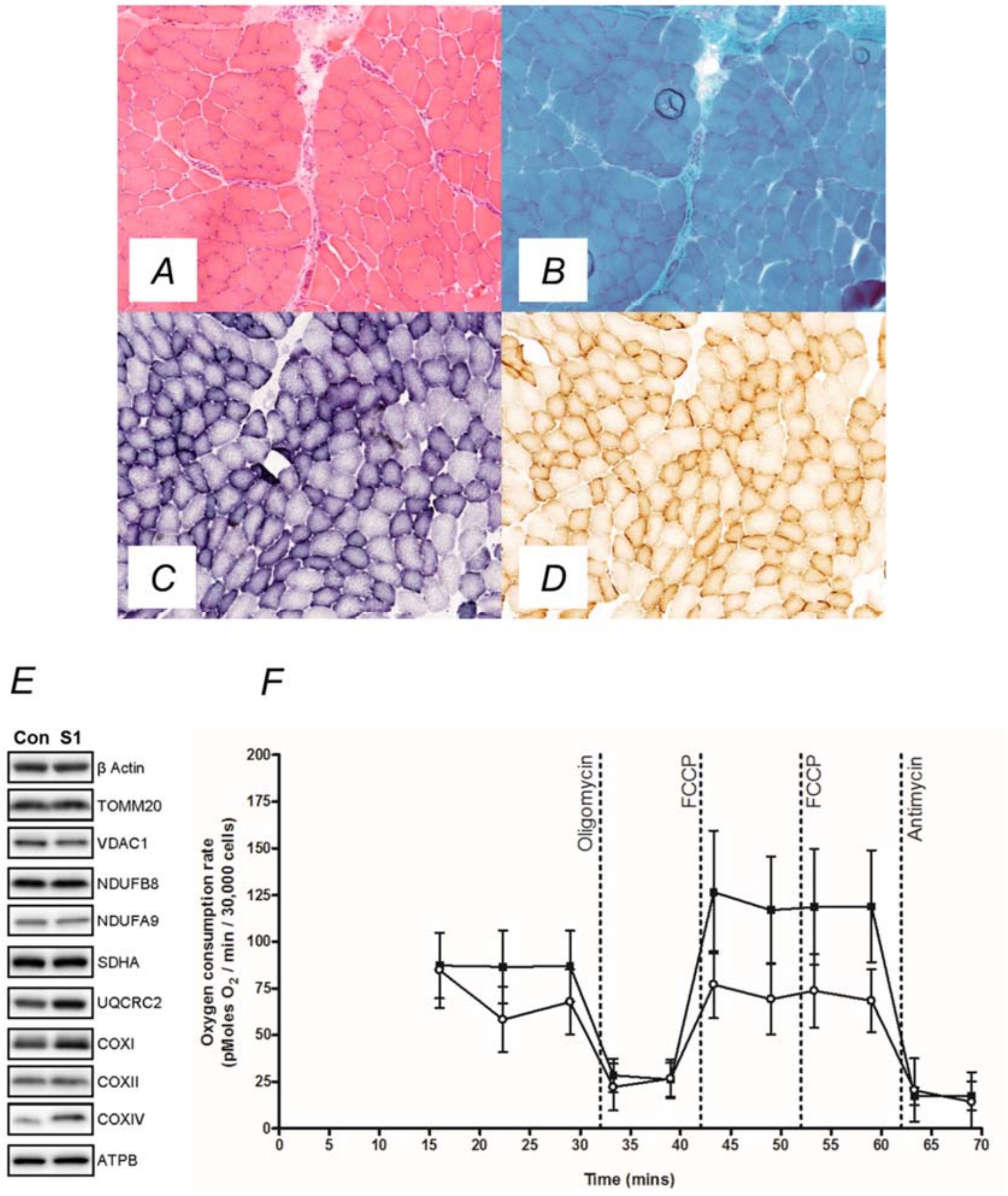

Figure S1: Functional characterization of muscle and cells from Subject 1
Histopathological analysis of a serially-sectioned skeletal muscle biopsy from Subject 1 (homozygous p.(Gly212Val) *TMEM126B* variant) showing (***A***) H&E staining, (***B***) modified Gomori Trichrome staining, (***C***) succinate dehydrogenase (SDH) and (***D***) cytochrome *c* oxidase (COX) reactions highlighting evidence of subsarcolemmal mitochondrial accumulation. Interestingly, subject fibroblasts did not show a significant OXPHOS defect, either based on immunoblotting of fibroblast mitochondrial proteins for OXPHOS components (***E***) or micro-scale oxygraphy analysis (Subject 1, n=10, white circles) compared to the combined data of control cell lines (n=5, black squares; Experimental details are described in detail previously[1]) although overall rates of oxygen consumption did appear to be generally decreased (***F***). Error bars indicate the standard deviation.

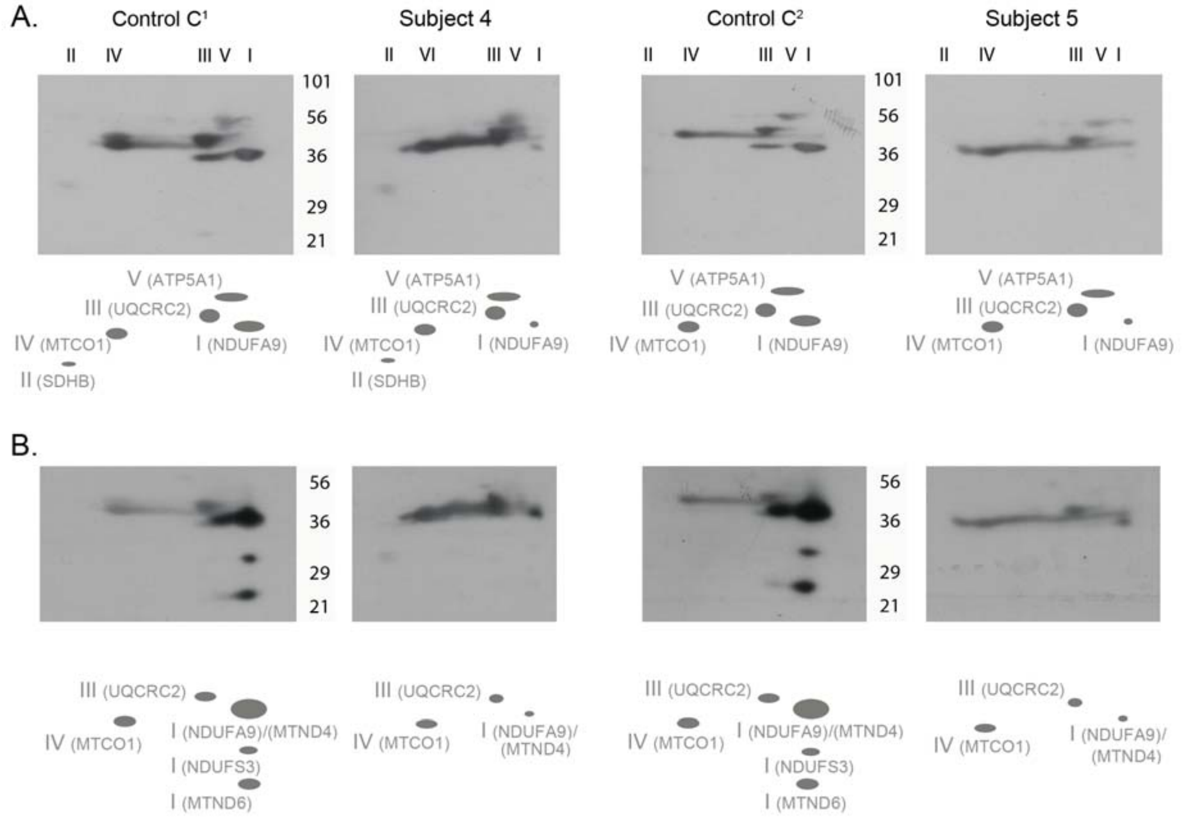

Figure S2: Western blotting following 2D BN-PAGE/tricine SDS-PAGE separation of isolated skeletal muscle mitochondria from Subjects 4 and 5
Experiments were performed according to the procedures previously described in detail[2]. (***A***) A mixture of the following antibodies was used to evaluate the abundance of the five OXPHOS protein complexes: complex I (NDUFA9), complex II (SDHB), complex III (UQCRC2), complex IV (MTCO1) and complex V (ATP5A1). (**B**) Following stripping of the antibodies, the nitrocellulose blot was reprobed using antibodies for complex I (MTND4, NDUFA9, NDUFS3 and MTND6), for complex III (UQCRC2) and for complex IV (MTCO1). An almost complete absence of signal with antibodies directed to the different complex I subunits is observed in skeletal muscle mitochondrial isolates from Subjects 4 and 5, highlighting a severe disturbance in the assembly of this OXPHOS complex.

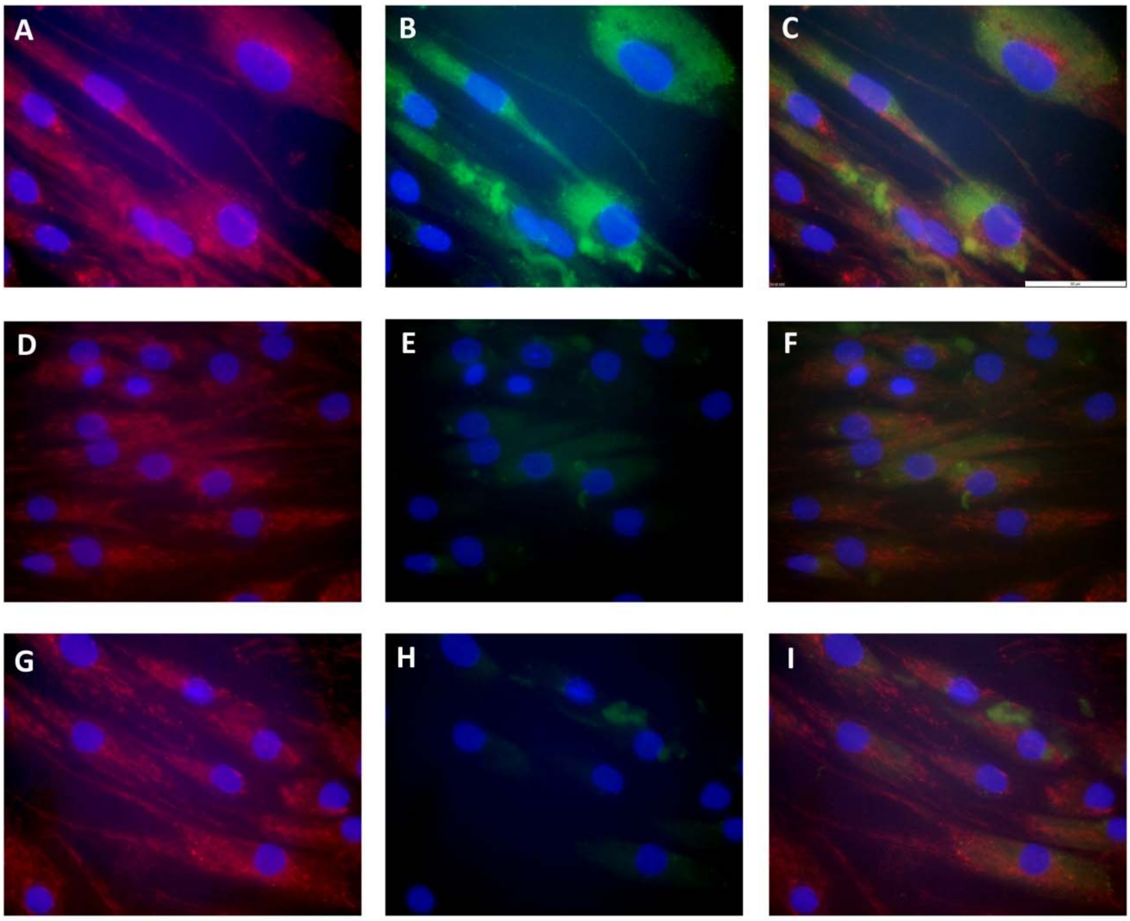

Figure S3: Cultured skin fibroblasts from Subjects 4 and 5 show severely decreased TMEM126B immunofluorescence

Double immunofluorescent staining of fibroblasts[3] from a control (***A-C***) and from Subject 4 (***D-F***) and Subject 5 (***G-I***) was performed, using MitoTracker Red CMXRos (Invitrogen) shown in red (panels ***A***, ***D*** and ***G***) and rabbit polyclonal anti-TMEM126B (AV49321, Sigma; 30µg/ml 2h room temperature) visualized with donkey anti-rabbit AlexaFluor488 (Invitrogen) shown in green (panels ***B, E*** and ***H***). Cell nuclei were counterstained with dapi shown in blue. The overlays (panels ***C, F*** and ***I***) demonstrate a reduction of TMEM126B staining in cells from both subjects (Scale bar = 50um).

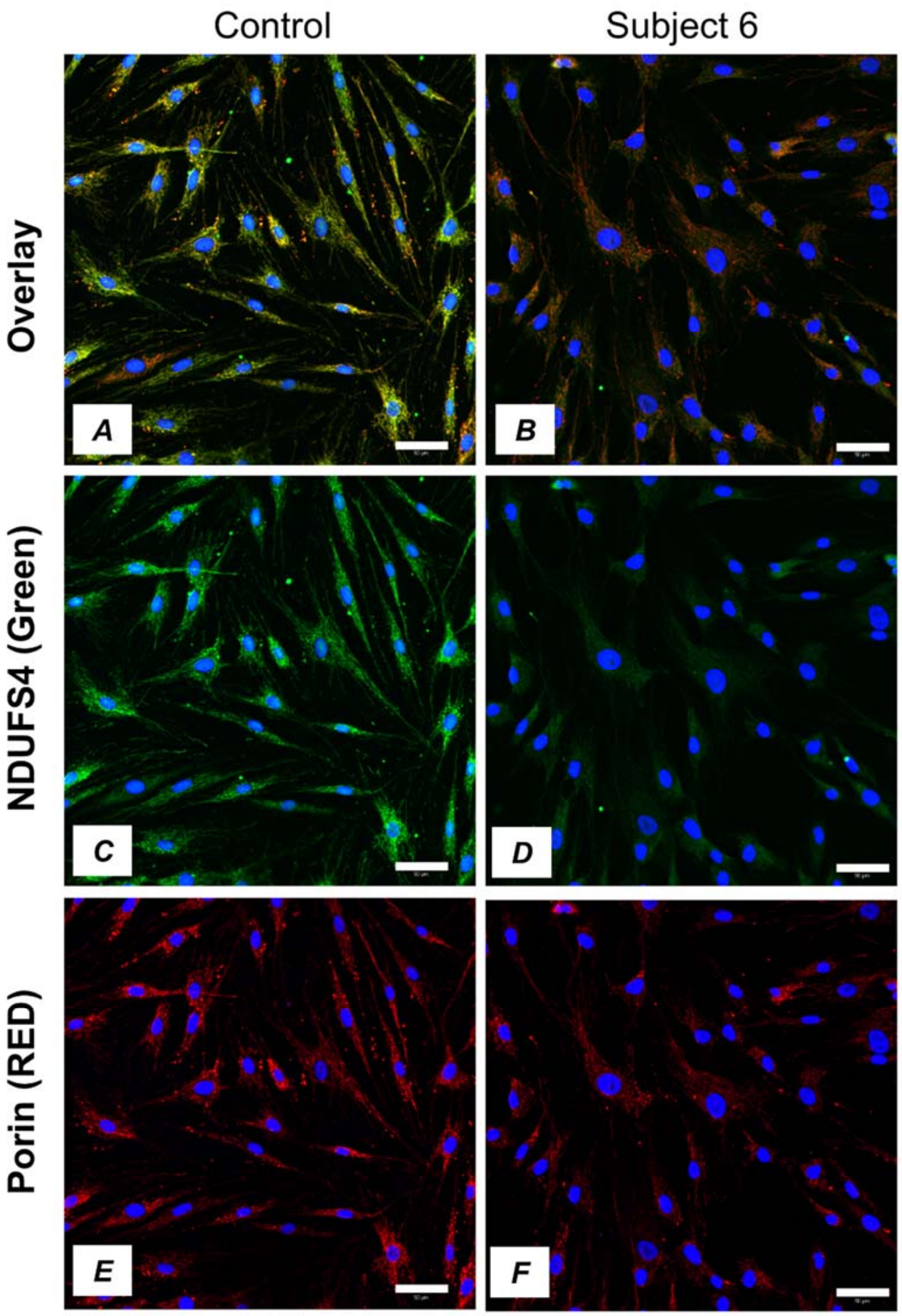

Figure S4: Cultured skin fibroblasts from Subject 6 express a complex I defect
Immunofluorescence staining of fibroblasts obtained from Subject 6 (homozygous p.(Gly212Val) *TMEM126B* variant) and a control was performed as reported [4] using the following primary antibodies: mouse monoclonal anti-NDUFS4 antibody (1:100; Abcam, Cambridge, UK) shown in green (panels ***C*** and ***D***) and rabbit polyclonal anti-VDAC1 (1:500; Abcam, Cambridge, UK) shown in red (panels ***E*** and ***F***), with the overlay (panels ***A*** and ***B***) clearly demonstrating strong staining of the NDUFS4 (complex I) protein in the control and absence in the patient cell line (Scale bar = 50um).

References

1. Bonnen, P.E., Yarham, J.W., Besse, A., Wu,P., Faqeih, E.A., Al-Asmari, A.M., Saleh, M.A., Eyaid, W., Hadeel, A., He, L., et al. (2013). Mutations in *FBXL4* cause mitochondrial encephalopathy and a disorder of mitochondrial DNA maintenance. Am J Hum Genet 93, 471-481.
2. van der Westhuizen, F.H., Smet, J., Levanets, O., Meissner-Roloff, M., Louw, R., Van Coster, R., and Smuts, I. (2010). Aberrant synthesis of ATP synthase resulting from a novel deletion in mitochondrial DNA in an African patient with progressive external ophthalmoplegia. J Inherit Metab Dis 33 Suppl 3, S55-62.
3. De Paepe, B., Smet, J., Vanlander, A., Seneca, S., Lissens, W., De Meirleir, L., Vandewoestyne, M., Deforce, D., Rodenburg, R.J., and Van Coster, R. (2012). Fluorescence imaging of mitochondria in cultured skin fibroblasts: a useful method for the detection of oxidative phosphorylation defects. Pediatric research 72, 232-240.
4. Ahting, U., Mayr, J.A., Vanlander, A.V., Hardy, S.A., Santra, S., Makowski, C., Alston, C.L., Zimmermann, F.A., Abela, L., Plecko, B., et al. (2015). Clinical, biochemical, and genetic spectrum of seven patients with NFU1 deficiency. Front Genet 6, 123.
